# Modulating Endoplasmic Reticulum Stress in Gastrointestinal Cancers: Insights from Traditional Chinese Medicine

**DOI:** 10.3390/ph17121599

**Published:** 2024-11-27

**Authors:** Qinyi Li, Xiaohong Zhao, Huan Yang, Xiaolong Zhu, Xinbing Sui, Jiao Feng

**Affiliations:** School of Pharmacy, Hangzhou Normal University, Hangzhou 311121, China; liqinyi2000620@163.com (Q.L.); zhaoxiaohong27@163.com (X.Z.); yhnpc1029@163.com (H.Y.); 2023112025090@stu.hznu.edu.cn (X.Z.); hzzju@hznu.edu.cn (X.S.)

**Keywords:** endoplasmic reticulum stress, unfolded protein response, traditional Chinese medicine, gastrointestinal tumors

## Abstract

Endoplasmic reticulum (ER) stress and the unfolded protein response (UPR) play critical roles in tumorigenesis, cancer progression, and drug resistance. Persistent activation of the ER stress system enhances the survival capacities of malignant tumor cells, including increased proliferation, invasion, and resistance to treatment. Dysregulation of ER function and the resultant stress is a common cellular response to cancer therapies and may lead to cancer cell death. Currently, growing evidence suggests that Traditional Chinese medicine (TCM), either as a monotherapy or in combination with other treatments, offers significant advantages in preventing cancer, inhibiting tumor growth, reducing surgical complications, improving drug sensitivity, and mitigating drug-induced damage. Some of these natural products have even entered clinical trials as primary or complementary anticancer agents. In this review, we summarize the anticancer effects of TCM monomers/natural products on the gastrointestinal (GI) tumors and explore their mechanisms through ER stress modulation. We believe that ongoing laboratory research and the clinical development of TCM-based cancer therapies hold considerable potential for advancing future cancer treatments.

## 1. Introduction

GI cancers represent the predominant cause of newly diagnosed cancer cases and cancer-related mortality on a global scale.

**Figure 1:**
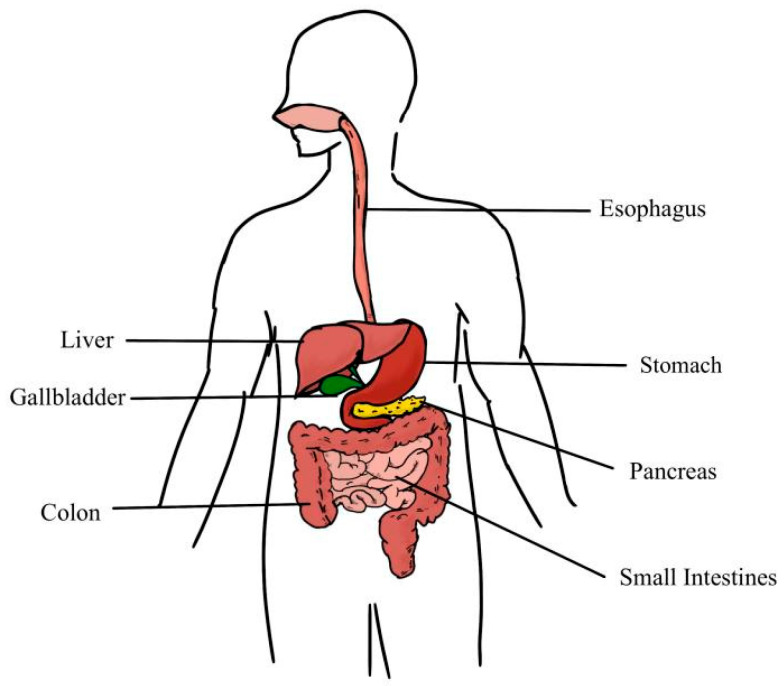
The human GI system.

Malignant cancers of the digestive tract encompass both epithelial and non-epithelial cancers, including esophageal, gastric, hepatocellular, colorectal, pancreatic, small intestine, bile duct, and gallbladder cancers [1]. GI cancers account for more than half of all cancer cases and deaths, making them the most common and deadly type of malignant cancer. Among them, colorectal, liver, and gastric cancers are particularly prominent, ranking among the top six cancers in both incidence and mortality globally. Colorectal cancer (CRC) ranks third in incidence and second in mortality among all cancers (Figure 1) [2]. Nevertheless, the treatment of GI cancer appears to have reached a bottleneck at this stage.

Treatments for GI cancers include surgery, radiotherapy, chemotherapy, targeted therapy, immunotherapy, and endoscopic resection [3,4,5,6]. Common chemotherapy drugs are 5-fluorouracil (5-FU), irinotecan, oxaliplatin, and paclitaxel. While effective, these drugs can damage normal cells and cause significant side effects such as nausea, vomiting, diarrhea, hair loss, and bone marrow suppression. Dosages must be carefully managed to balance efficacy and toxicity. In addition, resistance to chemotherapy over time is also a major challenge. Therefore, given the high degree of malignancy and mortality of GI cancer, and considering that existing drugs can no longer meet the progress of clinical treatment, it is urgent to develop new types of drugs.

TCM offers potential benefits in improving quality of life, preventing recurrence, prolonging survival, and enhancing survival rates for cancer patients. Recent research has highlighted the anticancer effects of TCM compounds such as artemisinin (ARS) [7], emodin [8], and berberine (BBR) [9,10]. These compounds can modulate signaling pathways to inhibit tumor growth, induce apoptosis, and reverse drug resistance.

ER is the primary site within cells for protein synthesis and processing, Ca^2+^ storage, and the metabolism of lipids and carbohydrates. When cells experience stress, such as hypoxia, nutrient deprivation, or disruptions in Ca^2+^ homeostasis, the functionality of the ER may become dysregulated. This leads to the accumulation of unfolded or misfolded proteins within the ER lumen, resulting in a pathological state known as ER stress. The cells initiate various adaptive mechanisms to restore protein homeostasis. These mechanisms include the UPR, ER-associated degradation (ERAD), and autophagy [11]. Prolonged or excessive ER stress can lead to apoptosis, while appropriate ER stress can help tumor cells survive and adapt to stress [12,13].

The aim of this study is to evaluate the efficacy of TCM monomers/natural products in the treatment of digestive system tumors through the modulation of endogenous ER pathways. While various studies have shed light on the anti-cancer mechanisms of these TCM monomers/natural products, research in this field remains fragmented, often consisting of isolated studies lacking coherence and systematic analysis. Thus, a comprehensive and systematic retrospective analysis is necessary. Herein, we highlight experimental approaches and key explorations in this research area using drug structure types as the framework and summarize the research of related nanoparticles and the natural products that have entered clinical trials. We provide new insights and entry points for laboratory investigations and the clinical development of cancer therapies based on Chinese medicine.

## 2. TCM as an Adjunctive Cancer Treatment

Currently, chemotherapy, radiotherapy, targeted therapy, and immunotherapy are commonly used to treat patients with intermediate and advanced malignancies. However, these treatments often come with severe side effects and numerous limitations. Decades of research have revealed that certain herbs can significantly reduce cancer-related pain, decrease the incidence of myelosuppression, and alleviate peripheral neuropathy and GI side effects. They also mitigate serious side effects specific to certain drugs, such as anthracycline-induced cardiotoxicity and radiation-induced pneumonia [14]. These toxicities not only severely impact patients’ quality of life but can also lead to discontinuation of treatment due to intolerance.

With advancements in medicine and increased awareness, cancer treatment has entered a stage of diversified and comprehensive approaches. TCM, considered a natural gift, is increasingly showing promising therapeutic effects as an adjunctive treatment for cancer patients worldwide. Compared to chemotherapeutic drugs, herbal compounds are relatively less toxic, more effective, and offer advantages such as multi-targeting. Reports suggest that TCM combined with chemotherapy or radiotherapy has significant benefits, including inhibiting tumor progression, alleviating surgical complications, enhancing the sensitivity of chemotherapy and radiotherapy drugs, improving the immune system’s function, and reducing the damage caused by surgery, chemotherapy, or radiotherapy [15]. Additionally, Chinese medicines contain a diverse range of chemical compounds, including alkaloids, polysaccharides, glycosides, and flavonoids, which have various biological functions and a wide range of effects on both innate and adaptive immunity [16].

Given these advantages, there is an urgent need to develop more effective complementary therapies for cancer treatment. TCM/natural products hold great promise as potential options for improving cancer treatment outcomes.

## 3. ER Stress and the UPR

**Figure 2:**
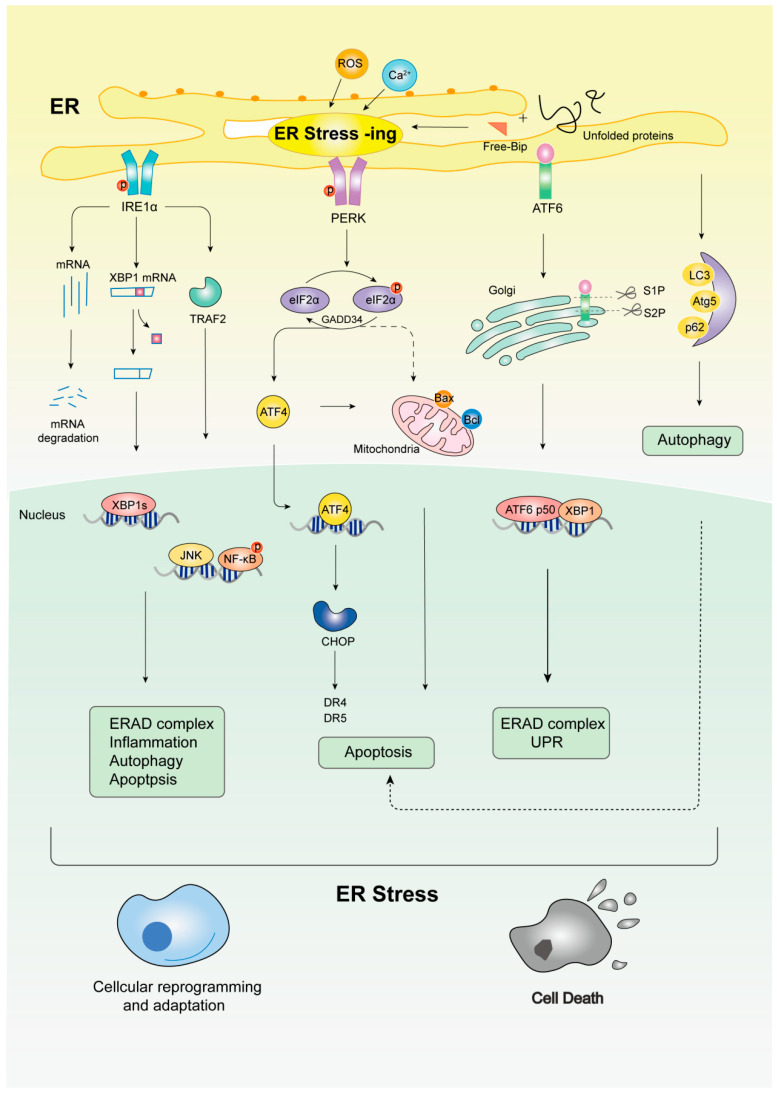
The mechanism of ER stress and UPR signaling pathways. Three UPR pathways are activated to restore ER homeostasis or trigger autophagy and apoptosis when the free Bip (GRP78) binds to PERK, IRE1, and ATF6 in response to an increase in unfolded/misfolded proteins brought on by a variety of factors, including hypoxia, vascular insufficiency, and nutritional deprivation.

ER stress triggers an adaptive response known as the UPR (Figure 2). The outcomes of UPR include the block of the protein translation, positive regulation of the protein folding-related molecular chaperones, and the upregulation of the signaling pathways responsible for targeting misfolded or unfolded proteins in the ER to mediate ubiquitin-mediated degradation. This response helps restore ER homeostasis and enhances cell survival under adverse conditions. If ER stress persists, the UPR can induce apoptosis.

The UPR is mediated by three major sensors located in the ER membrane: inositol-requiring enzyme 1α (IRE1α), activating transcription factor 6 (ATF6), and protein kinase R-like ER kinase (PERK). Under normal conditions, these sensors are bound to the molecular chaperone protein glucose-regulated protein 78 (GRP78/BiP), which inhibits their activation. When ER stress occurs, BiP preferentially binds to the misfolded proteins, releasing and activating the ER stress sensors and their downstream signaling pathways [17,18].

IRE1α is a bifunctional kinase and ribonuclease (RNase). Following UPR activation, IRE1α undergoes dimerization and autophosphorylation (phospho-Ser724 IRE1α). The active form of IRE1α induces the splicing of X-box binding protein 1 (XBP1) mRNA, resulting in the formation of the active XBP1-S isoform. XBP1-S positively regulates the expression of ER chaperones, genes encoding ERAD proteins, and lipid metabolism. Furthermore, IRE1α modulates the (c-Jun N-terminal kinase) JNK, nuclear factor kappa-B (NF-κB), and p38 mitogen-activated protein kinase (MAPK) signaling pathways, influencing inflammation, autophagy, and apoptosis [19,20].

ATF6 is a transmembrane glycoprotein and transcriptional activator that plays a crucial role in initiating the UPR signaling during ER stress. Upon sensing misfolded proteins, the full-length ATF6 (p90) is transported to the Golgi apparatus for processing and cleavage [21]. The cleaved p90 releases the N-terminal, cAMP-dependent form of ATF6α (p50) into the cytoplasm. Subsequently, p50 translocates to the nucleus where it binds to the ER stress response element (ERSE) on DNA, regulating the expression of ERAD and UPR genes [22].

PERK is a type I transmembrane protein acting as a sensor to regulate global protein synthesis. During the ER stress response and the activation of the UPR, PERK functions to inhibit the translation of new proteins. Activated PERK phosphorylates eukaryotic initiation factor-2α (eIF2α), thereby suppressing protein translation to maintain cellular homeostasis. This triggers the activation of transcription factor 4 (ATF4), a key adaptor that triggers genes related to amino acid metabolism, autophagy, oxidative stress response, and protein folding. However, prolonged translational blockade due to sustained PERK signaling can adversely affect cell viability. Growth arrest and proteins that cause DNA damage are upregulated by ATF4, and if ER stress remains unresolved, sustained PERK activation results in the production of C/EBP homologous protein (CHOP) [23].

## 4. ER Stress and Cancer

ER stress is closely related to cancer and has a dual effect on cancer. On one hand, under moderate stress, ER stress and UPR promote tumor cell survival through adaptive mechanisms. On the other hand, under severe or prolonged stress, ERS can induce apoptosis, suppressing tumor growth. The uncontrolled growth of malignant cells within tumors generates a challenging microenvironment marked by elevated metabolic demands, nutrient deprivation, hypoxia, and acidosis. These factors disrupt Ca^2+^ and lipid balance across various cell types in this environment. Consequently, the ER’s ability to fold proteins is impaired in cancer cells as well as immune cells, leading to an accumulation of misfolded or unfolded proteins, triggering ER stress. Oncogenic processes in cancer cells further intensify this issue by accelerating transcription and translation. In response, the UPR is activated, aiming to restore ER homeostasis and help cells adapt to the stressors present in the tumor. Thus, the ER coordinates a complex response to external stimuli, promoting tumor growth by activating adaptive mechanisms that sustain cellular survival and proliferation [24]. ER stress and UPR response are present in many types of cancer [25,26], including GI cancers [27,28,29], and ER stress is closely linked to the biological behaviors of malignant cancer and influences the proliferation and development of cancer to a large extent [30]. The activation of UPR signaling pathways is associated with several malignant biological behaviors, including proliferation, apoptosis, metastasis, angiogenesis, drug resistance, immune response, and resilience to treatments. Orchestrating ER stress responses is a highly dynamic process that could result in both pro-survival and pro-apoptotic outputs, which is largely depended on the intensity and duration of the UPR. When ERS exceeds the cell’s adaptive capacity, UPR shifts to a pro-apoptotic mode, activating apoptosis pathways (such as CHOP and JNK), leading to cell death. This occurs when the adaptive capacity of the UPR is overwhelmed and irreparable damage accumulates. Targeting ER stress pathways is an emerging cancer therapeutic strategy.

**Figure 3:**
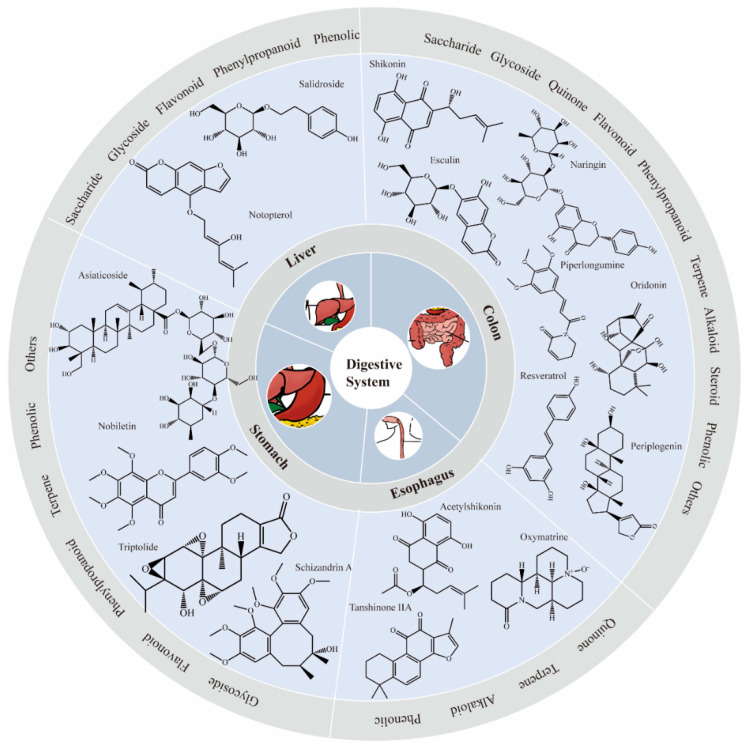
Representative TCM monomers and their classifications that exert therapeutic effect on GI cancers by stimulating ER stress.

Natural compounds have garnered significant interest in cancer treatment due to their diverse biological activities and relatively low toxicity compared to conventional chemotherapeutics. They have shown the potential to modulate the balance between pro-survival and pro-apoptotic responses to ER stress in tumor cells to obtain antitumor activity. Some natural products can act as inhibitors of the UPR pathways, reducing the adaptive responses of tumor cells to ER stress, hindering their survival and growth. Some natural products can exacerbate ER stress, pushing the UPR toward pro-apoptotic pathways, especially in cases where the tumor cells’ adaptive capacity to ER stress is overwhelmed. This mechanism makes natural compounds promising candidates for targeted cancer therapies (Figure 3, Table 1).

## 5. Natural Products Inducing ER Stress in GI Cancer

**Figure 4:**
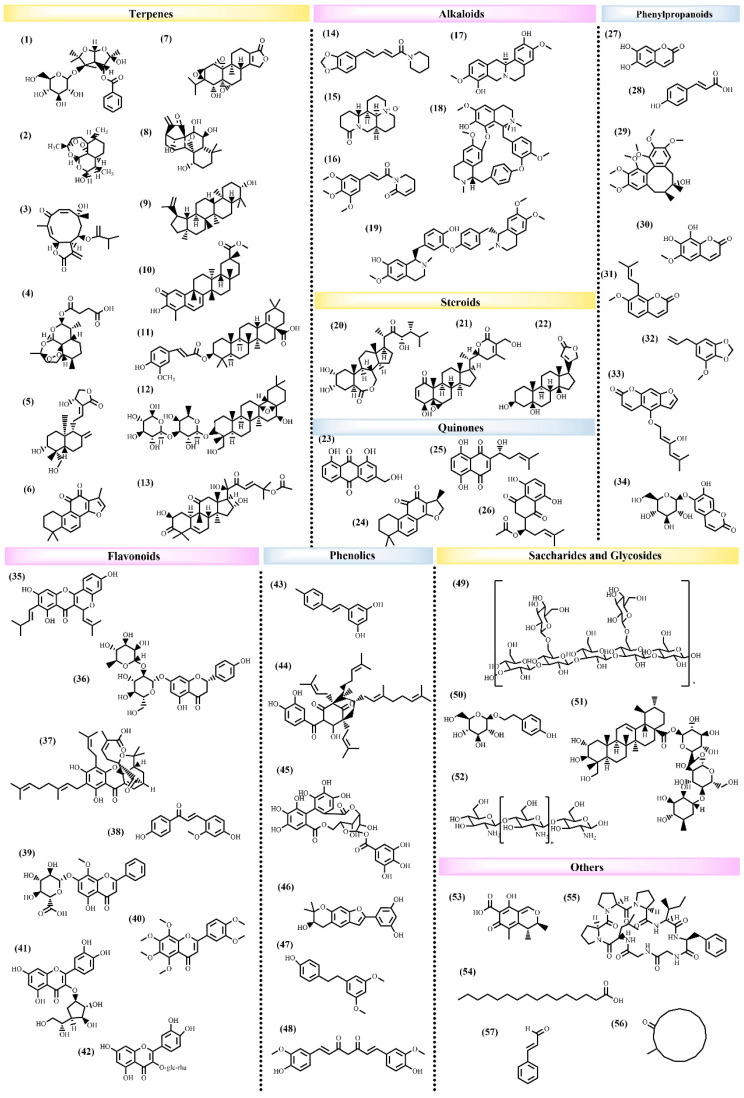
Structural formulas: TCM induces GI tumor cell death by regulating ER stress. (1) Paeoniflorin; (2) Dihydroartemisinin; (3) Tagitinin C; (4) Artesunate; (5) Andrographolide; (6) Tanshinone IIA; (7) Triptolide; (8) Oridonin; (9) Lupeol; (10) Pristimerin; (11) Glochodpurnoid B; (12)Saikosaponin A; (13)Cucurbitacin B; (14) Piperine; (15) Oxymatrine; (16) Piperlongumine; (17) Scoulerine; (18) Fangchinoline; (19) Daurisoline; (20) Epibrassinolide; (21) Withaferin A; (22)Periplogenin; (23)Aloe emodin; (24) Cryptotanshinone; (25) Shikonin; (26) Acetylshikonin; (27)Esculetin; (28) p-Coumaric acid; (29) Schizandrin A; (30) Fraxetin; (31) Osthole; (32) Myristicin; (33) Notopterol; (34) Esculin; (35) Brosimone I; (36) Naringin; (37) Gambogenic Acid; (38) Echinatin; (39) Wogonoside; (40) Nobiletin; (41) Isoquercitrin; (42) TTF1; (43) Resveratrol; (44) Oblongifolin C; (45) Corilagin; (46) Moracin P; (47) Pterostilbene; (48) Curcumin; (49) Lentinan; (50) Salidroside; (51) Asiaticoside; (52) Chitosan; (53) Citrinin; (54) Palmitic acid; (55) Heterophyllin B; (56) Muscone; (57) Cinnamaldehyde.

Recent studies have demonstrated that numerous natural products can exert antitumor effects by modulating ER stress, offering novel strategies for the treatment of GI tumors (Figure 4).

### 5.1. Saccharide and Glycoside

Saccharides can be classified as monosaccharides, disaccharides, and polysaccharides. Among them, polysaccharides are widely distributed in various plant tissues, including roots, stems, leaves, and fruits. Research has demonstrated that numerous polysaccharide compounds, such as those derived from angelica, astragalus, and ganoderma, exhibit a variety of bioactive effects. These include immunomodulatory effects, anti-tumor activity, anti-viral properties, the ability to lower blood sugar, and anti-aging benefits.

Natural polysaccharides represent a promising therapeutic approach for colon cancer, as they not only help maintain and enhance the balance of the intestinal microenvironment but also exhibit direct inhibitory effects on cancer cells. Lentinan (Compound **49**), a β-(1,3)-glucan polysaccharide isolated from *Lentinus edodes*, is widely considered to exert its antitumor effects indirectly by enhancing immune responses, particularly those mediated by T lymphocytes in the host [95]. Lentinan has been utilized as an adjuvant antitumor therapy for decades in the treatment of malignancies, including colorectal cancers. In vitro studies showed that lentinan could also directly attack cancer cells [96]. Zhang Y et al. discovered that lentinan exerts a nonimmune direct anti-tumor function against CRC cells. Both in vivo and in vitro experiments demonstrated that lentinan can disrupt the Ca^2^⁺ homeostasis and activate apoptosis and cytotoxic autophagy, both of which are influenced by the common upstream regulator of ER stress in a manner dependent on the PERK/ATF4/CHOP and IRE1α pathways [31].

Glycosides are composed of sugars and aglycones, which can include a variety of compounds such as alcohols, phenols, terpenes, and others. Glycosides exhibit a range of bioactive activities. For example, ginsenosides, rutin, and diosgenin have been found to possess multiple effects including anti-tumor, anti-fungal, antibacterial, antipyretic, sedative, and analgesic properties.

Salidroside (Compound **50**), a compound derived from *Rhodiola rosea* L., has demonstrated efficacy in inhibiting the progression of various tumors [97,98,99]. Shou-Yong Ding et al. reported that salidroside activates the PERK/eIF2α/ATF6 signaling cascade. Additionally, it was found to induce apoptosis in hepatocellular carcinoma (HCC) cells (HepG2) by promoting the release of cytochrome c into the cytoplasm, thereby activating caspase-8, caspase-9, and caspase-3. Furthermore, this process involves the regulation of Bax and Bcl-2 expression. And the knockdown of CHOP impairs the inhibitory effects of salidroside on HCC cells, suggesting that ER stress plays a crucial role in the cytotoxic effects of salidroside [32].

Asiaticoside (Compound **51**), a key compound isolated from *Centella asiatica* [100], was found to increase the expression of GRP78 and CHOP in GC cells, indicating that asiaticoside inhibits the proliferation and migration of GC by inducing ER stress. Furthermore, Zhang C et al. conducted a study on the expression of microRNA-635 (miR-635) in GC cells. The results indicated that the expression level of miR-635 in the control group of GC cells was significantly lower than that observed in the Asiaticoside treatment group. Furthermore, it was found that the expression of the miR-635 was elevated in the Asiaticoside group compared to that in the miR-635 inhibitor group. Therefore, asiaticoside can stimulate the ER stress of tumor cells by up-regulating miR-635 and inhibit the proliferation and migration of GC cells [33].

### 5.2. Quinone

Quinones are a general class of organic compounds that contain a conjugated cyclohexadiene–dione or cyclohexadiene–dimethylene structure. Quinones are widely distributed in nature, encompassing anthraquinones, naphthoquinones, benzoquinones, and phthalocyanines, and are involved in various biological processes, including antibacterial and antitumor activities.

Aloe emodin (Compound **23**), a rhodopsin extracted from *Aloe vera latex*, is an anthraquinone drug with antitumor effects against various tumors [101,102]. Aloe emodin treatment of CRC cells resulted in a significant increase in the expression of GRP78, PERK, and CHOP. Furthermore, PERK was found to promote the phosphorylation of eIF2α, as evidenced by the upregulation of calpain-1, calpain-2, and caspase-12 protein expression. These findings indicate that aloe emodin-induced ER stress leads to an excessive accumulation of cytoplasmic Ca^2+^, ultimately promoting cell apoptosis through the activation of the downstream effector protein caspase-12 [34].

Another TCM with antitumor effects in CRC is cryptotanshinone (Compound **24**), an important active ingredient derived from *Salvia miltiorrhiza* and classified within the naphthoquinone group. Wang et al. proposed that cryptotanshinone acts as an inducer of ER stress in a mouse model of colitis [103]. Additionally, Fu et al. demonstrated that the expression of Bip and phosphorylated PERK was significantly increased after treatment of cryptotanshinone in CRC cells. Furthermore, 4-phenylbutyric acid (4-PBA), a chemical chaperone able to alleviate ER stress by enhancing the folding capacity of proteins and promoting their proper processing within the ER, could reverse the cellular damage induced by cryptotanshinone. It was also hypothesized that the ER stress induced by cryptotanshinone was the upstream of autophagy, and that both ER stress and autophagy together contribute to the promotion of apoptosis [35].

Shikonin (Compound **25**), a naturally naphthoquinone derived from the roots of Lithospermum erythrorhizon, has been shown by Qi H et al. to exert anti-tumor effects through the activation of ER stress via the PERK/eIF2α/ATF4/CHOP and IRE1α/JNK signaling pathways. This ER stress activation leads to CRC cell death, mediated by the interplay of reactive oxygen species (ROS) generation, the mitochondrial apoptosis pathway, and ER stress-induced apoptosis [36]. Additionally, Zhang Z et al. demonstrated that the combination of shikonin with oxaliplatin enhances CRC sensitivity to oxaliplatin, with the proposed mechanism involving ROS-mediated activation of the ER stress pathway. This combination therapy holds promise for overcoming oxaliplatin resistance in clinical settings [37].

Acetylshikonin (Compound **26**), a fat-soluble naphthoquinone derived from *Lithospermum erythrorhizon* Sieb, exhibits anti-tumor, anti-inflammatory, and antibacterial properties. In a most recent study, Yuan YJ et al. found that acetylshikonin mediated apoptosis by inducing sustained ER stress and activated the PERK/eIF2α signaling pathway, leading to the upregulation of CHOP in oesophageal cancer cells. Inhibition of PERK or siRNA-mediated CHOP knockdown effectively rescued cells from acetylshikonin-induced apoptosis. These findings suggest that acetylshikonin holds potential as a promising candidate for the development of therapies targeting esophageal squamous cell carcinoma (ESCC). Additionally, the results of in vivo experiments also indicated the increased expression levels of BIP and CHOP in mouse tumor tissues [38].

### 5.3. Flavonoids

Flavonoids are a class of natural compounds with a C6-C3-C6 skeletal structure, widely found in plants and classified into various subtypes including dihydroflavonols, chalcones, and flavonols. These compounds exhibit numerous medicinal properties, including antioxidant, anti-inflammatory, antibacterial, immune-enhancing, and anti-tumor effects, making them highly valuable in nutraceutical, pharmaceutical, and cosmetic applications. Extensive research has been conducted on flavonoids, highlighting their significant potential, especially in natural product-based therapies.

Brosimone I (Compound **35**), an isoprenoid-substituted flavonoid from *Artocarpus heterophyllus*, was observed to induce ER stress in human colon cancer cells. This induction led to an increase in ROS production and cytoplasmic Ca^2+^ levels. Ultimately, the Ca^2+^/Calmodulin-dependent protein kinase kinase (CaMKKβ)- Adenosine 5‘-monophosphate (AMP)-activated protein kinase (AMPK) signaling pathway was found to be regulated, resulting in the inhibition of tumor cell development [39].

Gambogenic acid (Compound **37**), a flavonoid derived from Gamboge, has demonstrated anti-tumor activity across multiple cancer types. Liu C et al. demonstrated that gambogenic acid could induce ER stress in CRC via specifically inhibiting Aurora A kinase [40]. Aurora A kinase is frequently overexpressed in various types of cancers and plays a critical role in tumorigenesis and tumor development [104]. Aurora A was notably overexpressed in human colorectal adenocarcinoma tissue compared to normal epithelial cells. Gambogenic acid could induce ER stress, leading to the activation of the IRE1α and eIF2α pathways. Pretreatment with the eIF2α inhibitor salubrinal reduced the cell death caused by gambogenic acid. Additionally, gambogenic acid reduced Aurora A expression, and treatment with alisertib, an Aurora A inhibitor, alleviated ER stress. These results further imply that the inhibition of Aurora A kinase by gambogenic acid is beneficial for preventing the development of cancer.

Naringin (Compound **36**), a bioflavonoid derived from citrus fruits, was utilized in conjunction with tunicamycin and BAY 11-7082 (a NF-κB inhibitor) to effectively inhibit NF-κB and ROS-dependent PERK/eIF2α/ATF4/CHOP axis ER stress activation. Consequently, mitochondrial apoptosis of CRC cells was successfully induced [41]. In this investigation, CRC cells were not effectively inhibited by tunicamycin and BAY 11-7082 therapy alone. On the other hand, it dramatically boosted apoptosis when coupled with naringin. The expression levels of PERK/eIF2α/ATF4/CHOP axis were significantly increased when used in combination.

Echinatin (Compound **38**) is a type of chalcone flavonoid extracted from *licorice*. Ah-Won Kwak et al. investigated the effects of echinatin on CRC cells, including oxaliplatin-resistant strains. Echinatin could reduce the viability of both oxaliplatin-sensitive and -resistant CRC cells by generating ROS, which causes apoptosis and cell cycle arrest. The mechanism involves the activation of the JNK/p38 MAPK signaling pathway and increased ER stress. Inhibitors like n-acetylcysteine (NAC) and others were used to confirm that ROS and MAPK pathways are crucial for echinatin’s anticancer activity. This study highlighted echinatin as a potential therapy for drug-resistant colorectal cancer [42].

GC is also featured by high malignancy and poor prognosis. Flavonoids showing anti-tumor effects in GC cells include wogonoside, nobiletin, and isoquercitrin. Wogonoside (Compound **39**) is a natural flavonoid and the primary component of *Scutellaria baicalensis*. Wogonoside was found to reduce the viability of GC cells (AGS and MKN-45) and induce apoptosis through the activation of ER stress. Specifically, it triggered the IRE1α-TRAF2-ASK1 signaling pathway, leading to the phosphorylation of JNK and increased apoptosis. (TRAF2: tumor necrosis factor receptor-associated factors. ASK1: apoptosis signal regulating kinase-1.) Knockdown of IRE1α using siRNA prevented the apoptosis induced by wogonoside, confirming the pathway’s role in cell death. The findings highlight wogonoside’s potential as an anti-cancer agent by promoting ER stress-mediated apoptosis in GC cells [43].

Nobiletin (Compound **40**) is a flavonoid with notable efficacy and potential in inhibiting the growth of various tumors. It is widely found in citrus fruits, such as *Citri Reticulatae Pericarpium* [105]. *Citri Reticulatae Pericarpium* is one of the main components of Jianpi Yangzheng Xiaozheng (JPYZXZ), and it is the most often utilized qi-regulating herb in TCM, a decoction prescribed to patients with advanced GC. Chen et al. demonstrated that nobiletin significantly inhibits GC cell proliferation, induces apoptosis, and suppresses lipid metabolism by reducing de novo fatty acid synthesis. The mechanism involves the inhibition of key enzymes like atp citrate lyase (ACLY), acetyl coa carboxylase (ACACA), and fatty acid synthase (FASN), while also triggering ER stress via the IRE1α/GRP78/CHOP axis, which ultimately leads to cell apoptosis. Nobiletin treatment in mouse xenograft models confirmed its efficacy in reducing tumor size and weight. This research provides a potential therapeutic approach for GC by targeting lipid metabolism and ER stress pathways [44].

Isoquercitrin (Compound **41**) is a natural flavonol derived from quercetin, exhibiting higher bioactivity than quercetin. Its anticancer activity has been widely documented. Liu et al. discovered that isoquercitrin significantly inhibited the survival and proliferation of GC cells while inducing apoptosis in a dose-dependent manner. The mechanism involves the promotion of ER stress and the disruption of mitochondrial membrane potential, leading to cell death. And the ER stress inhibitor 4-PBA could reverse the effects of isoquercitrin. Isoquercitrin also enhanced the levels of immunogenic cell death (ICD) markers, such as calreticulin (CALR), adenosine triphosphate (ATP), high mobility group box 1 (HMGB1), heatshockprotein70 (HSP70), and heatshockprotein90 (HSP90), suggesting its potential role in immune system activation against cancer cells. These findings suggest that isoquercitrin is a potential therapeutic agent for triggering ICD [45].

### 5.4. Phenylpropanoid

Phenylpropanoids are a class of compounds characterized by a benzene ring linked to three straight-chain carbons (C6-C3 groups) and can be classified into simple phenylpropanoids, coumarins, and lignans. These compounds are widely distributed in nature, with examples including honokiol and dicoumarol.

Esculetin (Compound **27**) is a natural coumarin belonging to the phenylpropanoid group. Kim et al. discovered that esculetin induces ER stress, characterized by mitochondrial Ca^2+^ overload and upregulation of key ER stress-related proteins such as GRP-78, PERK, IRE1, and CHOP, leading to apoptosis. Additionally, esculetin was found to upregulate the expression of CHOP and caspase-12, further promoting ER stress-induced apoptosis in CRC cells [46].

Sharada H. Sharma et al. investigated the effects of p-coumaric acid (Compound **28**), a natural phenolic acid, on CRC cells. P-coumaric acid inhibits the proliferation of CRC cells by downregulating GRP78, a molecular chaperone involved in the UPR that is often upregulated in colon cancer [47]. By inhibiting Grp78, p-coumaric acid activates the PERK-eIF2α-ATF4-CHOP pathway, leading to UPR-mediated apoptosis in CRC cells. Additionally, p-coumaric acid can reduce inflammation by decreasing the expression of cytokines cyclooxygenase-2 (COX-2), interleukin- 6 (IL-6), tumor necrosis factor-α (TNF-α), and prostaglandin e2 (PGE2), as well as downregulating NF-κB signaling. These findings suggest that p-CA can target UPR signaling and inflammation, making it a promising anti-cancer agent for colon cancer therapy.

Schizandrin A (Compound **29**), a lignan isolated from *Schisandra chinensis*, belongs structurally to the phenylpropanoid group and has demonstrated anticancer effects [106]. Schizandrin A was found to inhibit the proliferation, invasion, and migration of GC cells. Upon schizandrin A treatment, the expression of Hsp70 member 5 (HSPA5), a marker of ER stress, increased in a concentration-dependent manner in AGS GC cells. Additionally, there was an increase in the phosphorylation of eIF2α and PERK, as well as elevated levels of CHOP, indicating that schizandrin A activated ER stress in AGS GC cells. Furthermore, 4-PBA was able to reverse schizandrin A-induced ERS, further confirming that schizandrin A induces ERS in AGS GC cells, thereby affecting cell proliferation and migration [48].

Fraxetin (Compound **30**) extracted from *Fraxinus* species has been shown to induce ROS production and Ca^2+^ influx, leading to a loss of mitochondrial membrane potential (ΔΨm) and ER stress. Additionally, fraxetin induces cell cycle arrest and modulates intracellular signaling pathways, including the PI3K/AKT and MAPK pathways. Notably, fraxetin’s inhibition of cell viability in CRC cells was found to be independent of the effects of 5-FU or irinotecan. This suggests that fraxetin could potentially serve as a primary or adjunct therapeutic agent targeting CRC in the future [49].

Osthole (Compound **31**), a type of coumarin, has been shown to promote the UPR and modulate the expression of p-eIF2α, ATF4, CHOP, and death receptor 5 (DR5) in CRC cells. Treatment with osthole resulted in enhanced sensitivity of these cells to cisplatin, along with increased expression of p-eIF2α [50].

Song et al. proposed that myristicin (Compound **32**) inhibits the EGFR/ERK signaling pathway, leading to reduced proliferation, increased apoptosis, and enhanced ERS in CRC cells. Additionally, treatment with myristicin resulted in an increase in cytochrome c release and mitochondrial ROS activity [51].

Notopterol (Compound **33**), the main bioactive component of *Notopterygium incisum* and a member of the furanocoumarins class, has been studied for its therapeutic effects and potential mechanisms in HCC. A study revealed that notopterol downregulated the expression levels of p-JAK2, ATF4, glutathione peroxidase 1 (GPX1), catalase (CAT), and superoxide dismutase 1(SOD1) proteins in liver cancer cell lines, thereby reducing the proliferation and invasion capabilities of cancer cells. Additionally, notopterol inhibited the cancer stem cell (CSC)-like phenotype in HCC cells and led to an increase in ROS production. In vivo experiments further demonstrated a significant increase in CHOP expression in mouse tumor tissues, indicating that notopterol induces ERS in tumor cells. In summary, notopterol shows promising clinical potential and may be developed as an effective ROS-mediated, CSC-targeted antitumor drug [52].

A most recent study has shown that esculin (Compound **34**), a bioactive compound from *Cortex fraxini*, induces ferroptosis (iron-dependent cell death) in CRC cells. This was evidenced by altered expression of key ferroptosis markers such as COX2, ASCL4, and ferritin heavy chain 1 (FTH1) following esculin treatment. The ferroptosis mode of cell death was further confirmed through the use of inhibitors, deferoxamine mesylate (DFO), and ferrostatin-1 (Fer-1). It is also hypothesized that ER stress may play a role in ferroptosis. Western blot analysis revealed that esculin modulates ER stress-related proteins, particularly PERK and phosphorylated eIF2α, and both in vitro and in vivo experiments confirmed that esculin induces ERS via activation of the PERK pathway. Additionally, esculin selectively activates the Nrf2/HO-1 pathway, contributing to CRC cell death [53].

### 5.5. Terpenes

Terpenes with the general formula of (C_5_H_8_)ₙ are a diverse group of naturally occurring compounds, including their oxygenated forms and varying levels of saturation. They are widely distributed in nature and are the key components responsible for the flavors, resins, and pigments of many plants. Terpenes are notably important in TCM due to their wide-ranging physiological effects, such as anti-inflammatory, anti-malarial, and anti-tumor activities. Several well-known terpenes serve as active ingredients in herbal medicines, with paclitaxel (used in cancer therapy) and artemisinin (used in malaria treatment) being prime examples. They are classified based on the number of isoprene units: monoterpenes (C_10_H_16_), sesquiterpenes (C_15_H_24_), diterpenes (C_20_H_32_), and so on. Terpenoids are modified terpenes that contain functional groups, typically oxygen-containing groups such as hydroxyl, carbonyl, or others.

Paeoniflorin (Compound **1**), a monoterpenoid derived from *Radix Paeoniae Alba*, has shown promising potential in cancer treatment. In a study by Kim TW et al., the knockdown of GRP78 in paeoniflorin-treated GC cells led to reduction in the expression of several key proteins involved in ER stress, including cleaved-caspase-3, CHOP, ATF4, p-PERK, and p-eIF2α. To explore how PERK signaling affects cell survival, PERK was knocked down. In AGS and SNU-638 GC cell lines, PERK knockdown alone did not prevent the paeoniflorin-induced reduction in cell viability and the increase in cleaved caspase-3, but CHOP knockdown did show a significant effect. Further investigation into the role of NADPH oxidase complex (Nox2 and Nox4) in paeoniflorin treatment revealed that Nox4 knockdown inhibited the paeoniflorin-induced reduction in cell viability and lactate dehydrogenase (LDH) levels [107]. It also led to a greater downregulation of cleaved caspase-3 and CHOP levels. These findings suggest that paeoniflorin exerts its effects on GC cells by regulating ROS production, Ca^2+^ release, and the ER stress response through increased Nox4 activity [54].

Dihydroartemisinin (Compound **2**) is a semi-synthetic compound derived from artemisinin, which has been shown to significantly increased the expression of GRP78 protein in CRC cells, which is associated with ER stress [108]. Dihydroartemisinin treatment significantly increased intracellular Ca^2^⁺ levels by inhibiting the activity of sarco/ER calcium ATPase (SERCA), leading to ER stress. Upregulation of CHOP activated the mitochondrial apoptosis pathway, as shown by increased Bax expression, decreased Bcl-2 levels, and activation of caspase-3. Silencing CHOP reduced dihydroartemisinin-induced apoptosis, indicating that ER stress plays a central role in dihydroartemisinin‘s anti-cancer effects, providing a potential therapeutic strategy for CRC treatment [55].

Tagitinin C (Compound **3**), a sesquiterpene lactone isolated from *Tithonia diversifolia*, has been shown to induce ferroptosis in CRC cells. Tagitinin C treatment increased the abundance of PERK, Nrf2, and HO-1. The reduction in cell death caused by PERK inhibitors suggests that PERK plays a key role in tagitinin C-induced ER stress, potentially through the activation of the Nrf2-HO-1 signaling pathway. Furthermore, it was demonstrated that tagitinin C and erastin, a known ferroptosis activator, synergistically induced ER stress, with the combined treatment group exhibiting the highest expression levels of PERK, BiP, Nrf2, and HO-1 proteins. This highlights the potential of tagitinin C to enhance ferroptosis via ER stress pathways [56].

Artesunate (Compound **4**) is derived from artemisinin, which is extracted from *Artemisia annua* L. A pilot trial that was double-blind, randomized, and placebo-controlled has demonstrated that artesunate exhibits anti-proliferative effects and is well tolerated in CRC patients. This study highlights the potential of artesunate as a therapeutic agent for CRC, showing promising results in inhibiting tumor growth while maintaining patient safety [109]. Regarding its mechanism, it is hypothesized that artesunate reduces cell viability by generating excess ROS. Artesunate treatment leads to the dissociation of IRE1α from BiP, followed by activation through autophosphorylation, both of which are significantly elevated at the protein level. Notably, both CHOP and its target protein DR5—an effector involved in eliciting the UPR and regulating apoptosis—are activated by artesunate. Thus, artesunate induces ER stress and activates the UPR via IRE1α signaling, ultimately leading to cellular autophagy [57].

Many chemotherapeutic drugs face the challenge of drug resistance, making combination therapy a more effective treatment strategy for tumors compared to monotherapy. Combining TCM with chemotherapeutic agents can provide significant advantages by addressing the limitations of each approach. Andrographolide (Compound **5**), a natural product, has been shown to increase ROS levels and induce ER stress, which is lethal to CRC cells [58]. In a study by Hong H et al., the combination of andrographolide with cisplatin demonstrated a synergistic effect, resulting in increased ROS accumulation and higher expression of p-eIF2α, ATF4, and CHOP compared to the control group or either treatment alone. Notably, silencing ATF4 inhibited cell death following combination treatment. Additionally, cells treated with both andrographolide and cisplatin showed significantly lower levels of phosphor signal transducer and activator of transcription 3 (p-STAT3) expression compared to those treated with andrographolide or CDDP alone. Thus, the combination of andrographolide and CDDP leads to tumor cell death through ROS accumulation, induction of ER stress, and inhibition of STAT3 phosphorylation [110].

Tanshinone IIA (Compound **6**) is a bioactive compound extracted from Danshen (*Salvia miltiorrhiza*). It has demonstrated significant efficacy in inducing apoptosis and inhibiting proliferation in human esophageal cancer Eca-109 cells in vitro. This anticancer effect is linked to the upregulation of cytochrome c and caspase-9 expression, which are critical components of the mitochondrial apoptosis pathway. Additionally, tanshinone IIA treatment leads to the downregulation of BiP, a chaperone protein associated with ER stress. These findings suggest that tanshinone IIA may exert its anticancer effects through both ER and mitochondrial pathways [59].

Triptolide (Compound **7**) is a natural bioactive compound extracted from the traditional herbal medicine *Tripterygium wilfordii*, known for its diverse biological activities, including anti-cancer properties. Currently, it is undergoing clinical trials to evaluate its effectiveness in cancer treatment. In triptolide-treated tumor cells, the expression levels of GRP78, phosphorylated eIF2α, ATF4, and CHOP were elevated, indicating that triptolide activates the eIF2α/ATF4/CHOP axis—an essential branch of the ER stress-mediated apoptosis pathway. Both the ROS inhibitor NAC and the ER stress inhibitor 4-PBA effectively reversed the triptolide-induced elevation of ER stress markers and apoptosis. Given that peroxiredoxin 2 (PRDX2) reduces ROS production and contributes to anti-apoptotic mechanisms in cancer cells, molecular docking techniques suggest that triptolide binds to PRDX2 at the Cys172 site, directly hindering PRDX2’s function. This interference may further impact the antioxidant and anti-apoptotic functions of PRDX2. Activity-based protein profiling (ABPP), a proteomic method that combines active molecular probe labeling with tandem mass spectrometry analysis, was used to identify PRDX2 as a direct binding target of triptolide. Triptolide enhances ROS generation by directly binding to and blocking PRDX2, which results in ER stress-mediated apoptosis [60].

Oridonin (Compound **8**), a diterpenoid compound with anti-tumor potential extracted from *Rabdosia rubescens*, has been demonstrated to trigger ER stress in CRC cells, as evidenced by increased levels of ATF4 and CHOP proteins. Through database analysis, three transcription factors related to disease-free survival in CRC patients were identified, potentially playing a role in regulating ER stress genes. Among them, Transcription Factor 4 (TCF4), which was sensitive to oridonin, was selected for further investigation using qRT-PCR. Target genes of TCF4 associated with survival in CRC patients, including cystic fibrosis transmembrane conductance regulator (CFTR), tyrosine-protein kinase Fyn (Fyn), and YOD1 deubiquitase (YOD1), were subsequently screened. The results demonstrated that both overexpression and knockdown of TCF4 significantly affected the expression of these target genes. In cell lines where TCF4 was knocked down, there was a notable increase in intracellular Ca^2+^ and ROS levels, as well as elevated expression of p-eIF2α, p-PERK, and CHOP. Previous reports suggest that the tumor-suppressive effect of tumor protein p53 (Tp53) is mediated by its disruption of the wingless-related integration site (Wnt)/β-catenin pathway, particularly by preventing TCF4 from binding to chromatin [111]. While the expression level of TCF4 was reduced in TP53 knockout tumor cells, the level of Wnt/β-catenin protein did not show a significant change. Therefore, it is speculated that oridonin may regulate the TP53/TCF4 axis by increasing ROS production and disrupting Ca^2+^ homeostasis, thereby inducing sustained ER stress and inhibiting tumor progression [61].

Lupeol (Compound **9**) is a pentacyclic triterpenoid phytosterol. A study highlighted lupeol’s potential in significantly reducing the viability of oxaliplatin-resistant CRC cells (LoVo). When treated with lupeol, these cells exhibited a decrease in the expression of ABCG2, an ATP-binding cassette efflux transporter associated with multidrug resistance (MDR). Additionally, levels of the ER stress marker p-eIF2α were notably elevated. In vivo animal experiments further demonstrated that lupeol effectively reduced the size of tumors transplanted from both oxaliplatin-sensitive and drug-resistant cell lines. These findings suggest that lupeol induces cancer cell apoptosis by inhibiting the ABCG2 protein and activating the ER stress pathway. Consequently, lupeol may represent a promising new strategy for the treatment of MDR cancers in the future [62].

Pristimerin (Compound **10**) is a natural quinonemethide triterpenoid. In CRC cells, pristimerin was found to promote the interaction between phorbol-12-myristate-13-acetate-induced protein 1 (Noxa) and Mcl-1, leading to increased apoptosis. Treatment with pristimerin resulted in the activation of ERS marker proteins and an increase in ROS production. However, NAC treatment effectively blocked pristimerin-induced ER stress and reduced Noxa expression, indicating that Noxa activation is particularly crucial in pristimerin-induced apoptosis. Overall, pristimerin induces Noxa expression by triggering the ROS/ER stress pathway, which promotes the formation of the downstream IRE1α-TRAF2-ASK1 complex and activates JNK [63].

Glochodpurnoid B (Compound **11**), a triterpenoid natural product obtained from the stems and branches of *Glochidion puberum*, has been shown to stimulate ER stress, leading to apoptosis. Treatment with glochodpurnoid B significantly altered the expression of both ER stress and apoptosis marker proteins. Notably, the knockdown of CHOP nearly abolished the expression of cleaved-PARP and cleaved-caspase-3, indicating a rescue from apoptosis. Additionally, the use of the 4-PBA inhibitor reduced the expression of these apoptosis marker proteins, further confirming the involvement of ER stress in the apoptotic process induced by glochodpurnoid B [64].

Saikosaponin A (Compound **12**), a triterpenoid glycoside isolated from *Bupleurum falcatum* L., has been investigated for its potential to overcome radioresistance in GC by targeting the PERK-ATF4-CHOP pathway within the ER. Previous experiments demonstrated that the saikosaponin A-induced decrease in cell viability, as well as the increase in LDH release and caspase-3 activity in GC cells, could be mitigated by the use of PERK inhibitors I and II. Moreover, saikosaponin A treatment was shown to elevate the levels of ER stress markers (GRP78, p-PERK, PERK, p-eIF2α, eIF2α, ATF4, and CHOP) in a time-dependent manner. The inhibition of ER stress signaling was found to block saikosaponin A-induced apoptotic cell death. In radioresistant GC cells, both saikosaponin A treatment and the combination of saikosaponin A with 2 Gy irradiation led to decreased cell viability and increased LDH cytotoxicity, with combined treatment showing a more pronounced effect than either treatment alone. While 2 Gy irradiation alone did not produce significant effects, the combined treatment upregulated E-cadherin levels and downregulated Slug and Snail in radioresistant cells, suggesting that saikosaponin A induces cell death by inhibiting the EMT in these cells. Further investigation using stable PERK knockdown cells revealed that treatment with saikosaponin A alone, 2 Gy irradiation alone, or their combination (saikosaponin A/2 Gy) did not result in significant changes in cell viability, LDH cytotoxicity, caspase-3 activity, ROS release, or Ca^2+^ release. Additionally, there were no observed effects on p-PERK, CHOP, or cleaved caspase-3 levels, indicating that PERK plays a crucial role in mediating the effects of saikosaponin A [65].

Cucurbitacins are a class of natural tetracyclic triterpenoids known for their various biological activities. The mechanism of action of cucurbitacin B (Compound **13**) primarily involves the activation of the ERS pathway. Treatment with cucurbitacin B led to the upregulation of key signaling pathway, specially the PERK/eIF2α/ATF4 and IRE1/XBP1 pathways, along with increased expression of ER stress markers such as GRP78 and CHOP. Furthermore, pretreatment with the ROS inhibitor NAC significantly attenuated cucurbitacin B’s effects on these proteins, suggesting that ROS-mediated activation of ER stress is crucial for inducing apoptosis in CRC cells. This indicates a potential therapeutic role for cucurbitacin B in targeting ER stress pathways in cancer treatment [66].

### 5.6. Alkaloids

Alkaloids are a diverse class of nitrogenous alkaline organic compounds found in various plants, typically characterized by complex ring structures with nitrogen atoms often integrated within the ring. These compounds exhibit a wide range of biological activity, making them significant active ingredients in herbal medicine. Alkaloids, such as camptothecin and atropine, are recognized for their anticancer, analgesic, and antibacterial effects, contributing to their therapeutic use in treating various ailments, and they continue to be a focus of research in pharmacology and medicinal chemistry.

Piperine (Compound **14**), a piperidine alkaloid found in black pepper, was shown to inhibit the growth of CRC cells (HT-29). Piperine induced G1 phase cell cycle arrest by decreasing the expression of cyclins D1 and D3 and by increasing levels of p21/WAF1 and p27/KIP1. Additionally, piperine treatment led to the production of hydroxyl radicals, loss of mitochondrial membrane integrity, and activation of apoptosis pathways, which were partially dependent on ROS. Futhermore, treatment with piperine resulted in an increased expression of ER stress-related proteins, including IRE1α, CHOP, and BiP, in HT-29 cells [67].

Oxymatrine (Compound **15**) is a quinolizidine alkaloid derived from the root of *Sophora flavescens*. Cao et al. treated xenograft EC in nude mice with oxymatrine injections. The treatment led to a significant reduction in tumor volume. Additionally, the expression levels of BiP were significantly decreased and CHOP was upregulated following treatment [68].

Piperlongumine (Compound **16**), a naturally occurring compound in *Piper longum* L., exhibits significant anticancer activity and functions as a direct inhibitor of thioredoxin reductase 1 (TrxR1). It exerts its effects in a ROS-dependent manner, leading to damage in malignant cells [112]. Due to the non-specific nature of chemotherapeutic agents, the doses used in treatment are typically low to minimize damage to normal cellular tissues. Consequently, combination therapy is often necessary in chemotherapy. In CRC cells, the IC_50_ of piperlongumine is three to four times higher than in normal intestinal cells. However, combining piperlongumine with oxaliplatin (Oxa) significantly increases ROS levels in tumor cells. Furthermore, levels of p-eIF2α, ATF4, and CHOP are markedly elevated following the combined oxaliplatin/PL treatment compared to either monotherapy (oxaliplatin or PL alone). This synergy between piperlongumine and oxaliplatin enhances anti-tumor activity, offering a novel approach for CRC treatment [69].

Scoulerine (Compound **17**) is an isoquinoline alkaloid isolated from *Corydalis* plants, which exhibits potent cytotoxic effects against tumor cells. Scoulerine was found to inhibit CRC cell viability in a dose-dependent manner, inducing apoptosis and increasing caspase-3/7 activity. Additionally, scoulerine triggered oxidative stress and ER stress in CRC cells, as evidenced by increased ROS, reduced glutathione, and upregulation of GRP78 and CHOP, with blocking ROS production or inhibiting ER stress mitigating the its effects on cell viability and apoptosis [70].

Fangchinoline (Compound **18**), an isoquinoline alkaloid, has been shown to significantly raise the expression of proteins linked to ER stress in CRC cells, such as p-PERK, p-eIF2α, ATF4, and CHOP. Furthermore, the knockdown of ER stress or using the inhibitor 4-PBA or CHOP prevented fangchinoline-induced apoptosis [71].

Daurisoline (Compound **19**), an isoquinoline from the *Rhizoma Menispermi*, has been shown to upregulate CHOP’s levels of both protein and mRNA. Alongside increasing concentrations of daurisoline, an upregulation of ATF4 protein was also observed. Knockdown experiments revealed that reducing the expression of ATF4 and CHOP largely reversed the cell death induced by daurisoline. Additionally, the downregulation of ATF4 significantly inhibited the expression of CHOP, Noxa, and DR5. These results show that daurisoline activates the p-eIF2α-ATF4 signaling axis to cause Noxa-dependent intrinsic apoptosis and CHOP-DR5-dependent extrinsic apoptosis, a process essential for the overaccumulation of ROS and activation of ER stress [72].

### 5.7. Steroids

Steroids, also known as steroid hormones, encompass a diverse class of natural compounds, including cardiac glycosides, steroidal saponins, and steroidal alkaloids. These compounds exhibit strong anti-inflammatory and immunomodulatory effects.

An earlier study focused on epibrassinolide (Compound **20**), a member of the brassinosteroid class of phytohormones which are naturally occurring polyhydroxy steroids. A study based on Stable Isotope Labeling by Amino acids in Cell culture (SILAC) data identified significant alterations in CALR expression following epibrassinolide treatment. CALR, a crucial protein in ER, plays a key role in UPR and Ca^2+^ buffering. Disruptions in CALR may trigger ER stress and UPR, leading to apoptosis. Epibrassinolide therapy induces ER stress and UPR by modulating CALR expression, ultimately resulting in caspase-dependent apoptosis in CRC cells. Notably, fetal human CRC cells and mouse embryonic fibroblasts treated with epibrassinolide did not exhibit UPR, indicating that epibrassinolide induces ER stress and apoptosis specifically in CRC cells without harming normal cells [73].

5-FU is a widely used chemotherapeutic agent for CRC; however, it is often associated with drug resistance and high toxicity. Withaferin A (Compound **21**) is a steroidal lactone from *Acnistus arborescens*. A study by Alnuqaydan AM et al. suggested that withaferin A holds promise as a complementary treatment to 5-FU for CRC. Their findings indicated that the combination therapy significantly promoted autophagy, as evidenced by the conversion of microtubule associated protein 1 light chain 3 beta-I (LC3B-I) to LC3B-II in CRC cells, as shown by Western blot analysis. Additionally, the expression of P62, a specific autophagy substrate, was notably elevated in the combination treatment compared to either withaferin A or 5-FU alone. The study further demonstrated that combination therapy led to a marked increase in ER stress markers, including BiP, CHOP, ATF-4, and the phosphorylation of eIF2α and PERK, with the effect being more pronounced in CRC cells. Rescue experiments validated these findings, and repeated knockdown studies concluded that the induction of apoptosis by the combination therapy was dependent on the PERK axis of ER stress, rather than the IRE1α or ATF-6 axes. Overall, combination therapy exhibited a potent synergistic effect, particularly by inhibiting the Wnt/β-catenin pathway, and offered the advantage of reducing the required therapeutic doses of both drugs [74].

Periplogenin (Compound **22**) is a natural compound from the traditional Chinese herb *Cortex Periplocae*. It has been reported to exhibit anti-inflammatory and anti-cancer properties. Yang Y et al. revealed that periplogenin induces ROS production in CRC cells and triggers apoptosis with mechanisms by modulating the BiP/eIF2α/CHOP and BiP/ASK1/JNK signaling pathways. Knockdown of CHOP or JNK proteins effectively blocks the pharmacological effects of periplogenin [75].

### 5.8. Polyphenols

Phenolic compounds encompass a broad spectrum of aromatic compounds characterized by the replacement of hydrogen on the aromatic hydrocarbon ring with a hydroxyl group (-OH). Polyphenol is a large class of naturally occurring phenols. Polyphenols hold significant pharmaceutical value due to their antioxidant, antibacterial, immune-boosting, and anti-cancer properties.

Resveratrol (Compound **43**), a well-known polyphenol, is an example. Over a decade ago, certain phenolic natural products, such as resveratrol, were identified for their anti-tumor activity through the modulation of ER stress markers, including p-eIF2α, CHOP, and GRP78 [76].

Oblongifolin C (Compound **44**), a phenolic compound derived from *Garcinia yunnanensis Hu*, revealed its ability to induce cysteine-dependent cell death in Bax/Bak-deficient cells, along with the concurrent induction of DNA damage and ER stress in both wild-type and knockout cells treated with oblongifolin C. Significantly increased phosphorylation of IRE1α and its substrate JNK1/2 suggests that oblongifolin C induces apoptosis by transcriptionally regulating the Bcl-2 family through CHOP [77].

Corilagin (Compound **45**) is a gallotannin from *Caesalpinia coriaria*. Wu C et al. demonstrated that corilagin could induce apoptosis, cell cycle arrest, and inhibit cell migration in EC cells. These effects were attributed to the activation of the mitochondrial pathway and the regulation of ER stress-related proteins both in vivo and in vitro. Specifically, corilagin treatment down-regulated the expression of GRP78 while up-regulating the expression of cleaved caspase-12 and -7 in EC cells [78].

Moracin P (Compound **46**), derived from *Morus alba* L. and identified among 30 extracts, is an active monomer characterized by its 2-arylbenzofuran structure. After treatment with it, most ER stress-related genes, such as baculoviral IAP repeat-containing 3 (BIRC3) and ATF4, were significantly upregulated in both cell lines studied. Gene Ontology analysis indicated that moracin P was enriched in pathways related to the ER stress response. Gene set enrichment analysis (GSEA) and Gene Set Variation Analysis (GSVA) further demonstrated that moracin P effectively activated unfolded protein responses and apoptotic pathways in HGC27 and MKN-45 cells. Terminal deoxynucleotidyl transferase (TdT) dUTP Nick-End Labeling (TUNEL) assay, commonly used to detect DNA fragmentation due to apoptotic signaling, revealed a significant increase in TUNEL-positive signals following moracin P treatment, indicating a notable rise in apoptosis rates. Subsequent in vivo experimental mechanistic studies confirmed this by showing significant upregulation of Bip and cleaved-PARP in tumor tissues [79].

Pterostilbene (Compound **47**), a natural stilbene abundant in blueberries, has demonstrated anti-cancer properties. In the context of utilizing phenolic compounds for tumor treatment, there is evidence of a pronounced synergistic anti-tumor effect when combined with commonly used chemotherapy agents in GC, such as lapatinib and doxorubicin (DOX), anticancer drugs that accumulated mitochondrial iron (II) (mtFe). Pterostilbene enhances the antitumor activity of low-dose sunitinib in GC cells by promoting the accumulation of mtFe, which in turn leads to increased mitochondrial H_2_O_2_ production. This induces ROS-associated hypoxia inducible factor-1α (HIF-1α) activation, causing ER stress and subsequent apoptosis, but not ferroptosis. Among the drugs tested, pterostilbene showed synergistic effects when combined with iron-accumulating drugs like DOX and lapatinib but not with 5FU or cisplatin. The results suggest that pterostilbene, when used with certain drugs that enhance mtFe accumulation, may offer novel therapeutic strategies for improving GC treatment. Further clinical investigation is needed to validate these findings [80].

Curcumin (Compound **48**), a diketone compound classified as a polyphenol, has been extensively studied for its anti-cancer properties. Early research demonstrated curcumin’s ability to impair mitochondrial function and induce RE stress in GC and CRC cells, positioning it as a potent therapeutic agent [81]. Subsequent studies showed that curcumin, when combined with chemotherapeutic agents like irinotecan, significantly enhanced anti-cancer effects by inducing ROS-dependent apoptosis and triggering ER stress, offering a more effective approach compared to single-agent therapies [82]. While the mechanisms underlying the curcumin-IRI combination remain underexplored, recent findings suggest that this combination upregulates ICD markers like CALR and HMGB1, further enhancing therapeutic outcomes in vivo. This has led to speculation that curcumin may promote a synergistic effect with irinotecan by inducing the ICD effect, potentially offering new insights and approaches for clinical drug use [83]. Recent advances indicate that ATF6 is a key target of curcumin during ERS, as curcumin activates ER stress by promoting ATF6 expression and its nuclear translocation, adding complexity to its mechanism of action, especially in combination therapies. This offers valuable insights into curcumin’s role in enhancing the efficacy of conventional chemotherapy [84].

### 5.9. Others

In addition to the clearly defined categories like alkaloids, phenolic compounds, and steroids, some compounds possess anti-cancer potential but do not fit neatly into any single classification. These compounds often exhibit complex structures or diverse mechanisms of action that transcend traditional categorizations. Their ability to induce apoptosis, inhibit proliferation, or trigger ER stress in cancer cells makes them valuable candidates in the search for new cancer therapies.

In the study by Tian PJ et al., X12-PG, a peptidoglycan derived from traditional cheese from Xinjiang, China, has shown potential anti-tumor effects in CRC. Peptidoglycan, a component of bacterial cell walls, is known to be an activator of the human immune response. The study suggests that X12-PG may harness this immune-activating property to exert its anti-tumor effects in CRC, providing a novel approach to cancer treatment [113]. Transmission electron microscopy (TEM) has revealed that X12-PG can significantly alter the morphology and structure of the ER, disrupting its physiological functions. This disruption leads to ER stress, which is induced by X12-PG treatment and subsequently promotes the release of Ca^2+^ from the ER into the cytoplasm. The elevated Ca^2+^ levels then serve as a signal that triggers the exposure of CALR on the cell surface and the release of HMGB1, both of which are key indicators of ICD. This suggests that X12-PG induces ICD, a form of cell death that can stimulate an immune response against cancer cells [85].

Citrinin, although primarily recognized as a mycotoxin responsible for food contamination and various toxic effects, has been reported to exhibit a broad spectrum of biological activities in vitro. Interestingly, despite its toxicity, citrinin has shown potential anticancer effects, making it a compound of interest in cancer research. This dual nature of citrinin highlights the complexity of its biological activity and suggests that, under controlled conditions, it might contribute to therapeutic strategies against cancer [114]. Citrinin has been observed to induce the production of ROS in CRC cells, leading to significant upregulation of ER stress markers such as GRP78 and GRP94. This upregulation suggests the occurrence of protein misfolding within the ER, triggering ER stress. Additionally, the elevated expression of growth arrest and DNA damage-inducible 34 (GADD34), protein disulfide isomerase family A member 6 (PDIA6), and the splicing of XBP1 mRNA indicates that citrinin activates the UPR. Notably, citrinin also induces the expression of CHOP, a critical pro-apoptotic factor, further implying that citrinin not only generates ER stress but also activates the UPR pathway in CRC cells (HCT116). This activation leads to cellular damage, which can be mitigated by treatment with the chemical chaperone PBA, highlighting the potential therapeutic implications of managing ER stress in citrinin-treated cells [86].

Palmitic acid, a long-chain saturated fatty acid commonly found in both animals and plants, has been shown to exert anti-tumor bioactivity through a mechanism involving ER stress. Palmitic acid influences intracellular iron levels, potentially by increasing cytoplasmic Ca^2+^ levels, which in turn regulate the transport of transferrin (TF), a key protein responsible for iron uptake. This ER stress-induced Ca^2+^ release appears to be a critical factor in this process. Additionally, it has been found that cells with high expression of CD36, a fatty acid transporter, are more sensitive to palmitic acid, suggesting that the effectiveness of palmitic acid in inducing ER stress and subsequent iron-related cell death may be influenced by CD36 levels. In summary, palmitic acid promotes anti-tumor activity by triggering ER stress, leading to ER Ca^2+^ release and TF-dependent ferroptosis (iron-dependent cell death) [87].

Heterophyllin B, a cyclic octapeptide isolated from *Pseudostellaria heterophylla*, has demonstrated significant anticancer activity in the context of GC. Studies have shown that heterophyllin B effectively hinders the proliferation of GC cells by activating ER stress and promoting apoptosis. In heterophyllin B-treated GC cells, there is a notable upregulation of key ER stress markers, including IRE1, CHOP, and GRP78. Concurrently, the expression of the anti-apoptotic protein Bcl-2 is down-regulated, further indicating that heterophyllin B induces apoptosis through the activation of ER stress pathways. This suggests that heterophyllin B could be a promising candidate for the development of new therapeutic strategies targeting GC [88].

Muscone has been investigated as a potential herbal medicine for the treatment of hepatocellular carcinoma in a study by Qi W et al. The expression levels of ATF4 and CHOP were significantly upregulated after muscone treatment. This research suggests that the pharmacological mechanism of muscone may converge on the PERK/ATF4/DDIT3 signaling pathway, ultimately inducing ER stress and apoptosis [89].

The signaling pathways that cinnamaldehyde acts on in GC cells are the PERK-eIF2α-ATF4-CHOP axis and the histone deacetylase/euchromatic histone lysine methyltransferase-2 (HDAC/G9a) pathway, ultimately mediating autophagic cell death. This provides a new layer of understanding of cinnamaldehyde [90]. Researchers initially established that cinnamaldehyde induces autophagic cell death and regulates cellular autophagic flux. They then investigated the relationship with ER stress and found that cinnamaldehyde treatment enhanced Ca^2+^ fluorescence intensity and increased the expression of GRP78, p-PERK, p-eIF2α, and CHOP. The results of knockdown reverse validation experiments pinpointed that the upstream cause of cell death is essentially ER stress.

### 5.10. Nanoparticle Drug Delivery Systems

Nanoparticle drug delivery systems have gained significant attention in recent years as a cutting-edge approach for cancer treatment. These systems offer a range of advantages, including enhanced bioavailability, targeted delivery, and improved efficacy of anticancer drugs. By encapsulating drugs within nanoparticles, it is possible to increase the concentration of the therapeutic agent at the tumor site while minimizing systemic toxicity and side effects. This targeted approach is particularly beneficial for delivering herbal medicines, which often face challenges related to bioavailability and stability. Nanoparticles can help overcome these limitations, making them a promising tool in enhancing the anti-tumor efficiency of herbal and conventional cancer therapies.

Additionally, nanoparticles can be engineered to have specific surface properties, allowing for the active targeting of tumor cells by recognizing and binding to specific receptors that are overexpressed on cancer cells. This targeted delivery not only improves the therapeutic index of the drugs but also reduces damage to healthy tissues, leading to better clinical outcomes. The ongoing research and development in nanoparticle drug delivery systems hold great promise for advancing cancer treatment, particularly in the integration of herbal medicines into modern therapeutic strategies [115].

5,2′,4′-Trihydroxy-6,7,5-trimethoxyflavone (TTF1) (Compound **42**) is the principal anticancer bioactive component of *Spiraea salicifolia* L. To overcome the challenges associated with the low absorbance and high biodegradability of TTF1, Xiao et al. developed nanoparticles of TTF1 (TTF1-NPs). Treatment with TTF1-NPs led to a concentration-dependent upregulation of GRP78, PERK, IRE1α, ATF6, caspase-4, and CHOP, which was inhibited by 4-PBA. These results imply that ER stress is a significant factor in mediating TTF1-induced apoptosis in HC [91].

Cinnamaldehyde is the main ingredient of cinnamon. In addition to the above application of cinnamaldehyde alone in GC treatment, it also has exploration clues in the field of nanoparticles. Cinnamaldehyde has been reported to have potent antitumor effects but has some limitations and drawbacks in clinical application due to aldehydes. Camptothecin has also been shown to have strong anticancer activity but low solubility in clinical trials; however, its derivative 10-hydroxy camptothecin (HCPT) has superior efficiency and toxicity. Based on these theoretical foundations, one study developed a novel self-assembled nanoparticle that can be applied to the synergistic therapy of CRC. Researchers attached cinnamaldehyde with biocompatible dextran and further constructed it with HCPT to form a PH-sensitive drug delivery system named HCPT-cinnamaldehyde-loaded nanoparticle (PCH) [92]. It was experimentally verified that both cinnamaldehyde alone and HCPT alone elevated ROS levels to some extent, whereas PCH caused a dramatic increase in ROS levels. Furthermore, time- and dose-dependently, PCH controlled the expression of proteins linked to ER stress, such as ATF4, p-eIF2α, and CHOP. Pretreatment with the ROS inhibitor NAC significantly reversed these changes. Further fluorescence imaging by in vivo experiments demonstrated that PCH accumulates predominantly in tumors and that the anti-tumor effect of co-treatment was more pronounced.

Chitosan is a natural polysaccharide with diverse applications. One study demonstrated that treatment of HCC cells with chitosan nanoparticles (CS NPs) resulted in significantly increased expression levels of cytochrome c and GRP78. This observation indicates the activation of both the mitochondrial pathway and ER stress, leading to substantial ROS production and ultimately apoptosis of HCC cells (SMMC-7721) [93].

Then, in another study, it can be seen that although there is a lot of research on curcumin, curcumin cannot make an outstanding contribution to the treatment of pancreatic cancer in the clinic at present, mainly because of the shortcomings of curcumin’s low bioavailability and instability [116]. New research intends to ameliorate the above problems using a material capable of sustained targeted drug delivery. Scientists proposed a method to dramatically improve the bioavailability of curcumin/gelatin-blended nanofibrous mats (Cc/Glt NMs) by electospinning of Cc/Glt NMs. It was verified that Cc/Glt NMs induced programmed cell death by activating the Bip/p-PERK/p-elF2α axis of the downstream ER stress signalling pathway. Significant effects were also observed in an in vivo animal model [94].

## 6. Advances in Clinical Trials of Natural Products in GI Treatment

Given the increasing potential of emerging anticancer therapies, there has been a growing interest in investigating TCM monomers and various natural products through clinical trials (Table 2). These compounds are being thoroughly evaluated not only for their potential in cancer prevention but also for their direct therapeutic effects in cancer treatment. Additionally, significant attention is being focused on their use as adjunct therapies, where they could increase the effectiveness of conventional treatments, lessen adverse effects, and overcome drug resistance mechanisms. The incorporation of these natural products into cancer treatment regimens offers a promising direction for personalized medicine and has the potential to optimize patient outcomes, marking an important step forward in oncological care.

## 7. Conclusions and Discussion

Herbal medicine plays a diverse and essential role in cancer care, offering symptom relief while also mitigating the adverse effects often caused by conventional treatments like chemotherapy, radiotherapy, and targeted therapy. By alleviating these side effects, particularly GI issues such as diarrhea, nausea, and vomiting, herbal medicine improves patients’ overall quality of life. It also shows promise in reducing complications like myelosuppression, chemotherapy-induced peripheral neuropathy, cardiotoxicity, and radiation-induced pneumonitis [117]. In addition, certain studies have indicated the beneficial impacts of Chinese herbal medicine on immune regulation. Herbal medicine can enhance the anti-tumor immune response by promoting the proliferation of T cells, increasing the proportion of tumor-killing macrophages and regulating the polarization of M2 macrophages. In recent years, it has also entered the stage of clinical trials, which shows that they are preparing for actual clinical treatment [118].

A thorough comprehension of the mechanisms of action and molecular targets of these herbal compounds is crucial for the development of innovative therapeutic strategies. Such knowledge enhances biomedical research by elucidating drug mechanisms, identifying the therapeutic potential of these compounds, and revealing target-independent side effects that may arise during treatment. Numerous studies have investigated the impact of herbal medicines on tumor progression, particularly in relation to ER stress, with a notable emphasis on the digestive system. This review aims to summarize the current findings regarding herbal medicines/natural products with ER stress-related mechanisms in GI tumors, providing new insights and potential therapeutic strategies for both monotherapy and adjuvant therapy in cancer treatment.

Despite the promising prospects of TCM/natural products as candidates for anticancer drug development, several challenges persist. These include poor water solubility, low stability, and limited specificity of many of these compounds. Moreover, the majority of these substances are still in the preclinical research phase, and the success rate of transitioning from laboratory findings to clinical application remains low [119]. Prior to clinical implementation, it is imperative to further investigate the efficacy, safety, and bioavailability of these compounds through rigorous testing in both animal models and human subjects. This underscores the notion that TCM still faces a considerable journey before it can be fully integrated into mainstream clinical practice. However, natural products, particularly those derived from TCM, have a solid foundation for future clinical application and should be continuously refined and innovated through the application of advanced scientific and technological approaches. Looking ahead, it is likely that a more sophisticated and comprehensive system of TCM for cancer treatment will emerge, further enhancing its role in modern oncology.

## Figures and Tables

**Table 1:** TCM induces the death of GI tumor cells by regulating ER stress.

Category	TCM	Cancer types	Mechanism	Ref.
Saccharide	Lentinan	CRC	p-IRE1α↑ p-PERK↑ p-eIF2α↑ ATF4↑ BIP↑ CHOP↑	[31]
			Bcl-2↓ Bax↑ Cleaved-caspase-3↑ p62↓ LC3-II↑	
Glycoside	Salidroside	HCC	p-PERK↑ p-eIF2α↑ ATF6↑ CHOP↑	[32]
			Bcl-2↓ Bax↑ Cyt-c↑	
	Asiaticoside	GC	CHOP↑ BiP↑	[33]
			E-cadherin↑ N-cadherin↓	
Quinone	Aloe emodin	CRC	BiP↑ p-PERK↑ p-eIF2α↑ CHOP↑	[34]
			Bcl-2↓ Bax↑ Calpain-1↑ Calpain-2↑ Caspase-12↑	
	Cryptotanshinone	CRC	BIP↑ p-PERK↑	[35]
			LC3B↑ Beclin-1↑	
	Shikonin	CRC	p-PERK↑ p-eIF2α↑ ATF4↑ BIP↑ CHOP↑	[36]
			Bcl-2↓ Cleaved-caspase-3/9↑ Cleaved-PARP↑ p-JNK↑	
	Shikonin+Oxaliplatin	CRC	p-PERK↑ p-eIF2α↑ ATF4↑	[37]
			Bcl-2↓ Bax↑ Cleaved-caspase-3↑ Cleaved-PARP↑	
	Acetylshikonin	ESCC	CHOP↑ BIP↑ p-eIF2α↑	[38]
			Bax↑ Cleaved-caspase-3↑ Cleaved-PARP↑	
Flavonoid	Brosimone I	CRC	p-PERK↑ CHOP↑ BiP↑	[39]
			CaMKKβ↑ p-AMPK↑	
	Gambogenic Acid	CRC	p-PERK↑ p-eIF2α↑ ATF4↑ BIP↑ IRE1α↑	[40]
			Aurora A↓	
	Naringin	CRC	p-PERK↑ p-eIF2α↑ CHOP↑ ATF4↑	[41]
			NF-κB↓ Bcl-2↓ Bax↑	
	Echinatin	CRC	CHOP↑ BiP↑ DR4↑ DR5↑	[42]
			Bcl-2↓ Bax↑ cyto-c↑ p-JNK↑ p-p38↑	
	Wogonoside	GC	CHOP↑ BiP↑ GRP94↑	[43]
			Bcl-2↓ Bax↑ Cleaved-caspase-3↑ p-ASK↑ p-JNK↑ TRAF2↑ IRE1α↑	
	Nobiletin	GC	CHOP↑ BiP↑	[44]
			Bcl-2↓ Bax↑ Cleaved-caspase-3↑ ACLY↓ FASN↓	
	Isoquercitrin	GC	p-PERK↑ p-eIF2α↑ CHOP↑ BiP↑	[45]
			Bcl-2↓ Bax↑ Cleaved-caspase-3↑ caspase-12↑ HMGB1↑ HSP70↑ HSP90↑	
Phenylprop-anoid	Esculetin	CRC	p-PERK↑ p-eIF2α↑ p-IRE1α↑ CHOP↑ BiP↑	[46]
			Cleaved-ATF6↑ Spliced XBP1↑ Cleaved-caspase-12↑	
	p-Coumaric acid	CRC	p-PERK↑ p-eIF2α↑ CHOP↑ BiP↑ ATF4↑	[47]
			Bcl-xL↓ cyto-c↑ P53↑	
	Schizandrin A	GC	p-PERK↑ p-eIF2α↑ CHOP↑	[48]
			MMP-2↓ MMP-9↓ E-cadherin↑ N-cadherin↓ Bcl-2↓ Bax↑ Cleaved-caspase-3↑ Cleaved-PARP↑	
	Fraxetin	CRC	BiP↑ CATF6α↑	[49]
			Bcl-2↓ Bax↑ MFN2↓ VDAC↓ p-AKT↑ p-ENK1/2↑ p-JNK↑ p-P38↑	
	Osthole	CRC	p-eIF2α↑ ATF4↑ CHOP↑ DR5↑	[50]
			Cleaved-caspase-3↑	
	Myristicin	GC	Bax↑ cyto-c↑ EGFR/ERK↓	[51]
	Notopterol	HCC	CHOP↑ ATF4↓	[52]
			p-Jak2↓ GPX1↓ CAT↓ SOD1↓ Snail↓ β-cat↓ N-Cad↓ OCT4↓ CD133↓ SOX2↓	
	Esculin	CRC	CHOP↑ BiP↑ p-eIF2α↑ PERK↑	[53]
			Bcl-2↓ Cleaved-caspase-3↑ P53↑ p-Nrf2↑ HO-1↑	
Terpene	Paeoniflorin	GC	p-eIF2α↑ p-PERK↑ ATF4↑ CHOP↑ BIP↑	[54]
			Cleaved-caspase-3/9↑	
	Dihydroartemisinin	CRC	CHOP↑	[55]
			Bcl-2↓ Bax↑ Caspase-3↑ Bid(MT)↑	
	Tagitinin C	CRC	BIP↑ IRE1α↑ PERK↑	[56]
			Nrf2↑ HO-1↑	
	Artesunate	CRC	BIP↑ IRE1α↑ p-IRE1α↑ CHOP↑ DR5↑	[57]
			LC3A↑ LC3B↑ p62↑	
	Andrographolide	CRC	p-eIF2α↑ ATF4↑ CHOP↑	[58]
			p-STAT3↓	
	Tanshinone IIA	EC	BIP↓	[59]
			cyto-c↑ Caspase-9↑	
	Triptolide	GC	CHOP↑ BiP↑ p-eIF2α↑ ATF4↑	[60].
			p62↓ LC3-II↑	
	Oridonin	CRC	CHOP↑ ATF4↑	[61].
			TP53↑ Wnt↓ β-catenin↓	
	Lupeol	CRC	p-eIF2α↑	[62].
			Caspase-3↑ ABCG2↓	
	Pristimerin	CRC	p-IRE1α↑ BiP↑ ATF6↑ p-PERK↑	[63]
			Cleaved-caspase-3↑ Noxa↑ p-JNK↑	
	Glochodpurnoid B	CRC	CHOP↑ ATF4↑	[64]
			Bcl-2↓ Bax↑ Cleaved-caspase-3↑ Cleaved-PARP↑	
	Saikosaponin A	GC	p-PERK↑ p-eIF2α↑ CHOP↑ ATF4↑	[65]
			E-cadherin↑ N-cadherin↓ Cleaved-caspase-3/8/9↑ Cleaved-PARP↑	
	Cucurbitacin B	CRC	p-PERK↑ p-eIF2α↑ BiP↑ ATF4↑ CHOP↑	[66]
			Bcl-2↓ Bax↑	
Alkaloid	Piperine	CRC	CHOP↑ IRE1α↑ BIP↑	[67]
			cyto-c↑ p-JNK↑ p38↑ p-Akt↓	
	Oxymatrine	EC	Bip↓ CHOP↑	[68]
	Piperlongumin	CRC	p-eIF2α↑ ATF4↑ CHOP↑	[69].
			p-JNK↑ Bcl-2↓ Bax↑	
	Scoulerine	CRC	CHOP↑ BiP↑	[70]
			Bcl-2↓ Bax↑ cyto-c↑	
	Fangchinoline	CRC	p-PERK↑ p-eIF2α↑ ATF4↑ CHOP↑	[71]
			Bcl-2↓ Bax↑ Caspase-3↑	
	Daurisoline	EC	p-eIF2α↑ CHOP↑ DR5↑ ATF4↑	[72]
			Noxa↑ P53↑ Puma↓ Bcl-2↓ Mcl-1↓	
Steroid	Epibrassinolide	CRC	p-eIF2α↑ CHOP↑ BiP↑ ATF6↑ ATF4↑	[73]
			CALR↑ Caspase9/12↓	
	Withaferin A	CRC	p-PERK↑ p-eIF2α↑ ATF4↑ CHOP↑ BiP↑	[74]
			Cleaved-PARP↑ LC3-II↑	
	Periplogenin	CRC	p-eIF2α↑ IRE1α↑ CHOP↑ BiP↑	[75]
			p-JNK↑ p-ASK1↓ Bcl-2↓ Bax↑ Cleaved-caspase-3↑ Cleaved-PARP↑	
Phenolic	Resveratrol	CRC	p-eIF2α↑ CHOP↑ BiP↑	[76]
			PARP↑ Caspase-3↓	
	Oblongifolin C	CRC	CHOP↑ IRE1α↑	[77]
			p62↓ LC3-II↑ Cleaved-caspase-3↑ Cleaved-PARP↑	
	Corilagin	EC	BiP↑	[78]
			Cleaved-caspase-3/7/8/9/12↑ MMP-2/9↓	
	Moracin P	GC	BiP↑ PERK↑ IRE1α↑ ATF6↑	[79]
			LC3-II↑	
	Pterostilbene	GC	CHOP↑ PERK↑	[80]
			Bcl-2↓ Bax↑ GPX4↑ HIF1α↑	
	Curcumin	CRC and GC	BiP↑ CHOP↑	[81]
			p-JNK↑ Fas↑ FasL↑ FADD↑ Bid↓ Bcl-2↓ Bax↑ cyto-c↑ Cleaved-caspase-3/7/8/9↑	
	Curcumin+Irinotecan	CRC	BiP↑ CHOP↑	[82]
			PDI↑	
	Curcumin	CRC	BiP↑ CHOP↑	[83]
			CAT ↑ HMGB1↑	
	Curcumin	CRC	BiP↑ CHOP↑ ATF6↑	[84]
			Bcl-2↓ Bax↑	
Others	X12-PG	CRC	Ca^2+^ ↑	[85]
			CAT↑ HMGB1↑	
	Citrinin	CRC	CHOP↑ BiP↑ GRP94↑ PDI6A↑ GADD34↑	[86]
			Bax↑	
	Palmitic acid	CRC	p-eIF2α↑ p-PERK↑ ATF4↑	[87]
			SOD2↑ GPX4↑ TFRC↓ TF↑ FPN↑	
	Heterophyllin B	GC	IRE1α↑ CHOP↑ BiP↑	[88]
			Bcl-2↓	
	Muscone	HCC	p-eIF2α↑ p-PERK↑ ATF4↑ CHOP↑	[89]
			Bcl-2↓ Bax↑ Cleaved-caspase-3↑ LC3-II↑ p-AMPK↑ p-mTOR↓	
	Cinnamaldehyde	GC	p-eIF2α↑ p-PERK↑ CHOP↑ BiP↑	[90]
			Bcl-2↓ Cleaved-caspase-3/9↑ ATG5↑ Beclin-1↑ p62↓ LC3B↑ p-mTOR↓ p-AMPKα↑ p-ULK1↑	
Nanoparticless	TTF1 NPs	HCC	CHOP↑ PERK↑ BiP↑ ATF6↑	[91]
			p-JNK↑ Caspase-4↑	
	Cinnamaldehyde NPs	CRC	p-eIF2α↑ CHOP↑ ATF4↑	[92]
			Bcl-2↓ Bax↑ P53↑	
	Chitosan NPs	HCC	BiP↑	[93]
			Bax↑ Cleaved-caspase-3↑ cyto-c↑	
	Cc/Glt NMs	PAAD	p-eIF2α↑ p-PERK↑ BiP↑	[94]
			Cleaved-caspase-3↑ p-STAT3↓	

Notes: CRC: colorectal cancer; HCC: hepatic cell carcinoma; GC: gastric cancer; EC: Esophageal cancer; ESCC: Esophageal squamous cell carcinoma; PAAD: Pancreatic adenocarcinoma.

**Table 2:** Clinical trials based on TCM alone and in combination in GI cancers.

Posted	Identifiers	TCM	Cancer	Phase	Notes
2004	NCT00094445	Curcumin	Pancreatic Cancer	II	-
2005	NCT00256334	Resveratrol	Colon Cancer	I	-
2005	NCT00192842	Curcumin+Gemcitabine	Pancreatic Cancer	II	-
2006	NCT00295035	Curcumin+Gemcitabine+Celebrex	Colorectal Cancer	III	-
2007	NCT00433576	Resveratrol	Colorectal Cancer	I	-
2007	NCT00486460	Curcumin+Gemcitabine+Celebrex	Pancreatic Cancer	III	-
2008	NCT00745134	Curcumin+Capecitabine	Colorectal Cancer	II	Radiotherapy
2009	NCT00973869	Curcumin	Colorectal Cancer	I	Prevention
2011	NCT01490996	Curcumin C3+ FOLFOX	Colorectal Cancer	I/II	Inoperable Patients
2011	NCT01294072	Curcumin	Colorectal Cancer	I	Plant Exosomes
2013	NCT01993472	Andrographolide+Capecitabine	Colorectal Cancer	II	-
2013	NCT01859858	Curcumin+Irinotecan	Colorectal Cancer	I	-
2014	NCT02195232	Isoquercitrin	Colorectal Cancer/Pancreatic Cancer	II/III	-
2014	NCT02261844	Resveratrol	Hepatocellular carcinoma	I/II	-
2015	NCT02439385	Curcumin+Avastin+FOLFIRI	Colorectal Cancer	II	-
2016	NCT02724202	Curcumin+5FU	Colorectal Cancer	I	-
2017	NCT03093129	Artesunate	Colorectal Cancer	II	pre-operation
2017	NCT03129139	Minnelide+Protein-Bound Paclitaxel	Gastric Cancer/Colorectal Cancer/Pancreatic Cancer	I	Pro-drug of triptolide
2019	NCT04196075	Andrographis Paniculata	Esophageal Cancer	III	Palliative Care
2021	NCT04896073	Minnelide	Pancreatic Cancer	II	Pro-drug of triptolide
2022	NCT05856500	Curcumin+creatine	Gastric Cancer/Esophageal Cancer	-	-
